# Will deep brain stimulation increase the incidence of induced psychosis? Post-operation follow-ups for 1 hundred patients from 2004-2017

**DOI:** 10.1051/bmdcn/2018080421

**Published:** 2018-11-26

**Authors:** Paul-Jer Chen

**Affiliations:** 1 Department of Neurosurgery, China Medical University Hospital, China Medical University Taichung 404 Taiwan

**Keywords:** Deep brain stimulation, Psychosis, Neurosurgery

## Abstract

Generally regarding as a safe treatment for Parkinson’s disease (PD) for the past 20 years, deep brain stimulation (DBS) is also an example of precision medicine where surgeons need to titrate individual patient’s stimulating electrodes one by one down to the scale of micrometer for the maximum therapeutic effect. In order to prevent operation induced psychiatric complications and minimize any other potential side effects, we have followed 103 patients received this treatment provided by a single surgeon in the same medical institution from 2004 to 2017. We identified each patient complaint from nursing care records and complication data from medical charts during the perioperative hospitalization period to see if any of them correlate statistical significantly with the DBS lead placement procedure. Top five frequent complaints including fever, constipation, nausea, headache, wound pain. The majority of post-operative complaints turned out to be the same as general post-operative / post-anesthesia side effects rather than the DBS operation itself. However, a few rare but critical complications such as post-operative intracranial hemorrhage (ICH), postoperative epidural hematoma (EDH) were identified as well. These patients’ subsequent treatments and prognosis were documented for revising the operating procedure in the future. Our retrospective study reconfirmed that DBS is indeed a relatively safe procedure and improve the life quality of PD patients in general. Hopefully, the through preoperative preparation and careful surgical approach will safeguard the patient’s prognosis.

## 1. Introduction

There are more than 80,000 patients worldwide receiving Deep Brain Stimulation (DBS) as treatment for Parkinsonian symptoms in the past two decades. Taking the advantage of DBS’ reversibility and titratability, the patients with DBS enjoy satisfiable living standard.[1–4] However, DBS is a costly procedure with its risk & concerns as well.[5–8] Here we look back at the 103 patients receiving this treatment by a single surgeon in the same medical institution from 2004 to 2017. We plan to quantified the in hospital complication / complaints to see if any of them correlate statistical significantly with the DBS lead placement procedure. The data we gathered will also serve us directions for revising the standard procedure in the future. The optimal stimulation location for therapeutic effect is individually determined for each case based on patient evaluation, preoperative MR images or CT scans, intraoperative microelectrode recording, and often, intraoperative direct stimulations.

## 2. Methods

The subjects used for the statistical analysis were collected from patients operated by Dr. Chou Shang-Ming during his time at China Medical University Hospital starting 2004. Total patients from this time period was 104, but 1 patient, who developed heart attack when getting imaging study, was not operated. The complications were recognized solely via the nursing care records and data from charts. The data is analysis with Excel® for the descriptive statistic and SPSS® for analytical statistics.

## 3. Results

The total number of subjects counted is 103; the great majority (~90%) of DBS target used is Subthalamic nucleus (STN). Globus Pallidus interna (Gpi) and Pedunculo-Pontin Nucleus (PPN) were also used in small amount of patients (~5% each) secondary to their specific symptoms. 57 patients are male, 46 patients are female. Male to female ratio is 55% to 45%. Age ranges from 33 to 80 years old. Male average 62.6 years old; female average age is 59.9. As summarized in Table 1, hospital course for DBS placement ranges from 10 to 33 days, after dropping one septic shocks patient stayed for 84 days and one psychosis patient stayed for 117 days. 18 cases had their DBS placement completed by single operation: Average hospital stay was 16.7 days. 85 cases had their DBS system placed at two different time periods. Inbetween operation days range from 2-12 days mainly for lead function testing. This group of patient stayed in the hospital for an average of 19.5 days. As far as time spending in the operating rooms goes, single stage surgery takes about 6.15 hours to complete, whereas two stage procedures require 5.13 hours for the first stage and 2.39 hours for the second stage.

**Table 1:** Basic information of patient group & hospital stay.

**Sex and age**		

Male	57(55%)	average 62.6years old
Female	46(45%)	average 59.9 years old
**One stage surgery (18 cases)**		

Operating time		6.15 hours
Average hospital days		16.7 days
**Two stages surgeries (85 cases)**		

First operating time		5.13 hours
Days between operation		average 5.1 days
Second operating time		2.39 hours Average
Hospital stay		19.5 days

Table 2 summarized the post operation complaints. For patients receive their DBS system in one single operation, GI symptoms such as nausea, constipation, headache, wound pain and fever are the most frequent complains in the chart. One patient developed septic shock and was re-intubated due to aspiration pneumonia. Other less frequent complaints includes transient muscle power drop, blurred vision, tinnitus, sore throat...etc. For patients going through stage surgeries, the first stage involves placing stimulating leads in the brain with wounds in the scalp. Fever is the highest complaints following by headache, wound pain, constipation, and insomnia. 2 patients were identified with delirium and then psychosis / hallucination but both patients’ symptoms recovered subsequently. For the second stage operation involving mostly neck and chest, wound pain, constipation, GU symptoms and fevers again are the most common complaints, followed by Nausea / vomiting, Insomnia, Tremor,...etc. Three new patients with delirium were recorded after the second half surgery. However, their symptom improved before discharge.

**Table 2:** In hospital adverse events after DBS operation.

**One stage surgery (18 cases)**		

Nausea / Constipation	11 / 18	patients (~61%)
Headache / Wound pain	7 / 18	patients (~39%)
Fever	6 / 18	patients (~33%)
**1 Shocked, reintubated due to aspiration pneumonia**		

Transient muscle power drop	2 / 18	patients (~11%)
Hiccup	2 / 18	patients (~11%)
Transient Diabetes incipidus	1 / 18	patients (~6%)
Blurred vision	1 / 18	patients (~6%)
Tinnitis	1 / 18	patients (~6%)
Sore throat	1 / 18	patients (~6%)
Insomnia	1 / 18	patients (~6%)
Psychosis / Delierium	0 / 18	patients (0%)
**Two stages surgeries (85 cases) After first operation**		

Fever	33 / 85	patients (~39%)
Headache / Wound pain	30 / 85	patients (~35%)
Nausea / Vomiting / Constipation	22 / 85	patients (~26%)
Insomnia	12 / 85	patients (~14%)
Tremor	11 / 85	patients (~13%)
GU Symptoms	6 / 85	patients (~7%)
Seizure	3 / 85	patients (~4%)
Transient muscle power drop	2 / 85	patients (~2%)
Depression	2 / 85	patients (~2%)
Shortness of breath	2 / 85	patients (~2%)
Delirium / Psychosis	1 / 85	patients (~1%)
Delirium / Audio hallucination	1 / 85	patients (~1%)
Epidural hematoma (EDH)	1 / 85	patients (~1%)
Tachecardia	1 / 85	patients (~1%)
**After second operation**		

Wound Pain	38 / 85	patients (~45%)
Wound poor healing, debrid	ement 3 / 85	patients (~4%)
Nausea / Vomiting / Constipation	23 / 85	patients (~27%)
GU symptoms	10 / 85	patients (~12%)
Fever	10 / 85	patients (~12%)
Insomnia	5 / 85	patients (~6%)
Tremor	5 / 85	patients (~6%)
Dilirium	3 / 85	patients (~4%)
Trasient muscle power drop	3 / 85	patients (~4%)
Fall	3 / 85	patients (~4%)
Shortness of breath	1 / 85	patients (~1%)
Transient blurred vision	1 / 85	patients (~1%)
Depression	1 / 85	patients (~1%)
Verbal confusion	1 / 85	patients (~1%)
Psychosis	1 / 85	patients (~1%)
Oligouria, GCS drop, UTI after 1^st^ stage		

## 4. Discussion

Previous studies pointed out that operation induced psychiatric complication can be observed within two weeks and usually present initially as delirium. Our data, on the other hand, shows that most complains are not different from those commonly observed after general anesthesia. No specific complications are attributed to the DBS procedure. This conclusion is based on the data that mostly gathered directly from the chart and nursing record, however, there could still be selective bias on what to be recorded by the health care personals on the day to day basis. Also, patients’ condition after discharged is not available in detail, which makes the long term data collecting less comprehensive.

However, a few rare but critical complications such as postoperative ICH, post-operative EDH did get identified in the process. These complications occurrence rate was not higher than what to expect after general neurosurgery operations,[9] but is serving to remind us that explanation before the operation must be through and complete since complications, serious or not, will shows up from time to time.[10]

The DBS is a costly procedure requires precision surgical techniques during the operation and up most care during the following perioperative period as well. Our study shows that DBS is indeed a relatively safe procedure. If the target selected carefully, the induced psychosis can indeed be minimized. The serious complications we ran into also provide us with opportunities to modify the lead placement procedure and re-evaluate the standard of care for the patient in the ward.[11] Hopefully, the through preoperative preparation and careful surgical approach will safeguard the patient’s prognosis.

## Conflict of interest statement

The author disclose no conflicts of interest.
